# The Long-Term Effects of Developmental Hypoxia on Cardiac Mitochondrial Function in Snapping Turtles

**DOI:** 10.3389/fphys.2021.689684

**Published:** 2021-06-28

**Authors:** Gina L. J. Galli, Ilan M. Ruhr, Janna Crossley, Dane A. Crossley

**Affiliations:** ^1^Faculty of Biology, Medicine, and Health, School of Medical Sciences, The University of Manchester, Manchester, United Kingdom; ^2^Developmental Integrative Biology Research Group, Department of Biological Sciences, University of North Texas, Denton, TX, United States

**Keywords:** heart, metabolism, reptile, developmental programming, reactive oxygen species, electron transport chain, oxygen affinity, developmental plasticity

## Abstract

It is well established that adult vertebrates acclimatizing to hypoxic environments undergo mitochondrial remodeling to enhance oxygen delivery, maintain ATP, and limit oxidative stress. However, many vertebrates also encounter oxygen deprivation during embryonic development. The effects of developmental hypoxia on mitochondrial function are likely to be more profound, because environmental stress during early life can permanently alter cellular physiology and morphology. To this end, we investigated the long-term effects of developmental hypoxia on mitochondrial function in a species that regularly encounters hypoxia during development—the common snapping turtle (*Chelydra serpentina*). Turtle eggs were incubated in 21% or 10% oxygen from 20% of embryonic development until hatching, and both cohorts were subsequently reared in 21% oxygen for 8 months. Ventricular mitochondria were isolated, and mitochondrial respiration and reactive oxygen species (ROS) production were measured with a microrespirometer. Compared to normoxic controls, juvenile turtles from hypoxic incubations had lower Leak respiration, higher P:O ratios, and reduced rates of ROS production. Interestingly, these same attributes occur in adult vertebrates that acclimatize to hypoxia. We speculate that these adjustments might improve mitochondrial hypoxia tolerance, which would be beneficial for turtles during breath-hold diving and overwintering in anoxic environments.

## Introduction

Low O_2_ availability (hypoxia) is a common environmental stressor in aquatic, terrestrial, and subterranean environments (Bickler and Buck, 2007). Hypoxia can have severe metabolic consequences for animals, because O_2_ plays a pivotal role in the production of ATP and reactive oxygen species (ROS) during oxidative phosphorylation (Mitchell, 2011). Briefly, electrons derived from carbohydrates, fats, and proteins are transferred to the mitochondrial electron transport chain, where they move through a series of complexes and finally bind to O_2_ at complex IV. The energy released from the transfer of electrons is utilized by complexes I, III, and IV to pump protons against their electrochemical gradient and establish a proton-motive force that drives ATP production through complex V (the F_1_F_o_ ATP-synthase) (Papa et al., 2012). Although most electrons complete this journey, a small proportion slip from the chain and bind directly to molecular O_2_ to produce superoxide (Brand, 2016). When O_2_ becomes limiting, the electron transport chain is inhibited and electron slip becomes more common, leading to reduced ATP production and the overproduction of ROS (Solaini et al., 2010). Consequently, prolonged periods of hypoxia are associated with energy depletion and oxidative stress, which can ultimately lead to cell death.

Despite the profound metabolic consequences of hypoxia, many animals successfully exploit hypoxic environments (Bickler and Buck, 2007). Ectothermic vertebrates are particularly hypoxia-tolerant, with many species inhabiting aquatic and subterranean environments, where O_2_ can become severely limited or even completely absent (anoxia) (Bickler and Buck, 2007; Richards, 2009). These organisms have evolved a suite of metabolic adaptations that compensate for O_2_ deprivation (Richards, 2009). For example, acclimatization to hypoxia is commonly associated with metabolic rate suppression, a downregulation of oxidative phosphorylation, and the activation of anaerobic pathways (Richards, 2009). Central to this strategy is a structural and functional remodeling of the mitochondria (Fuhrmann and Brune, 2017; Sokolova, 2018) that usually involves a reduction in mitochondrial content and a downregulation of enzymes involved in oxidative phosphorylation (Johnston and Bernard, 1982; Donohoe and Boutilier, 1998; St-Pierre et al., 2000a; Heather et al., 2012; Hickey et al., 2012; Murray and Horscroft, 2016). Although these modifications reduce ATP production, the strategy serves to limit ROS production and oxidative stress, which can have disastrous consequences for cellular survival (Brand, 2016). The adaptive nature of this response is also supported by studies that show mitochondrial content and aerobic capacity are reduced in species that are genetically adapted to hypoxia, such as human populations at high altitude (Horscroft et al., 2017) and hypoxia-adapted fly strains (Ali et al., 2012). To overcome the decline in oxidative capacity, some vertebrates improve the efficiency of oxidative phosphorylation by modifying aspects of the electron transport chain. For example, humans acclimatized to high altitude have lower levels of uncoupling proteins and proton leak, leading to an improvement in mitochondrial coupling efficiency (the amount of ADP phosphorylated per oxygen consumed) (Jacobs et al., 2012; Levett et al., 2012). Similarly, different isoforms of complex-IV subunits can be preferentially expressed under hypoxic conditions, resulting in a higher complex-IV turnover rate and reduced oxygen affinity (Sinkler et al., 2017; Pajuelo Reguera et al., 2020). In aggregate, these studies demonstrate that mitochondria are capable of remarkable phenotypic plasticity and play an essential role in hypoxic survival.

Although the effects of hypoxia on mitochondrial function in vertebrates are well studied in adulthood, far less is known about embryonic life stages. Among ectotherms, oviparous species are particularly prone to hypoxic episodes during embryonic development (Lutz and Dunbar-Cooper, 1984; Booth, 1998; Wu, 2009; Podrabsky and Wilson, 2016). Several studies have shown that chronic hypoxia leads to an active suppression of embryonic oxygen consumption in a range of ectotherms, including fish (Gnaiger et al., 1987; Miller et al., 2008; Lindberg and Di Giulio, 2019; Wood et al., 2019), frogs (Bradford and Seymour, 1988), turtles (Kam, 1993), and crocodiles (Booth, 2000). Furthermore, exposure to hypoxia increases embryonic hypoxia tolerance by lowering critical oxygen tensions (P_Crit_) in snapping turtles, zebrafish, and salmon (Kam, 1993; Barrionuevo et al., 2010; Del Rio et al., 2019; Wood et al., 2019). The reduction in embryonic metabolism and improvement in P_Crit_ could be driven, at least in part, by mitochondrial remodeling. Indeed, similar to adult vertebrates, chronic hypoxia leads to a reduction in embryonic mitochondrial content and aerobic capacity in zebrafish (Lindberg and Di Giulio, 2019; Laquatra et al., 2021), which might contribute to metabolic suppression. Importantly, mitochondrial remodeling during embryonic development could have profound consequences for ectothermic vertebrates, because adaptive responses during early life can often be permanent, leading to life-long changes in morphology, physiology, and behavior (West-Eberhard, 2003). Nevertheless, to our knowledge, only one study has investigated the long-term impact of developmental hypoxia on mitochondrial function in an ectothermic vertebrate. In that study from our laboratory, cardiac mitochondria from juvenile alligators that were previously exposed to developmental hypoxia had lower levels of proton leak and higher respiratory-control ratios, which indicate enhanced mitochondrial efficiency (Galli et al., 2016). This is an interesting finding, because it suggests that embryonic exposure to hypoxia can program mitochondrial traits in ectothermic vertebrates that might be beneficial for hypoxic survival in adulthood. To explore this hypothesis further, the present study investigated the long-term impact of developmental hypoxia on mitochondrial function in the common snapping turtle, *Chelydra serpentina*.

Snapping turtles bury their eggs in subterranean nests that regularly become hypoxic, due to the combined changes in environmental gas conductance, rising egg-mass metabolism, and the metabolic activity of microorganisms (Papa et al., 2012; Brand, 2016). The extent of hypoxia in turtle nests is variable and depends on nest shape and egg location, but some species can be subjected to levels of O_2_ as low as 11% of air saturation (Papa et al., 2012). Programming of hypoxia-tolerant traits would be particularly beneficial for snapping turtles, because this species regularly encounters hypoxic environments during adulthood, and they overwinter in anoxia for up to 5 months in ice-covered lakes (Ultsch, 2006). Importantly, laboratory studies have shown that juvenile and adult snapping turtles from hypoxic incubations have altered cardiac structure and function (Eme et al., 2013, 2014; Tate et al., 2015; Wearing et al., 2016, 2017), lower levels of basal ROS production, and improvements in cardiomyocyte anoxia tolerance (Ruhr et al., 2019). These changes are likely to involve adjustments in mitochondrial function. Therefore, we hypothesized that developmental hypoxia programs snapping turtle mitochondrial aerobic capacity and ROS production in a manner that protects the heart from hypoxic or anoxic injury. In support of our hypothesis, we found that developmental hypoxia improved the efficiency of ATP production, as well as reducing basal levels of ROS production.

## Materials and Methods

### Turtle Collection, Incubation, and Husbandry

Snapping turtle (*C. serpentina*) eggs were collected from the wild in Minnesota, United States, and transported to the University of North Texas for incubation (altitude elevation = 70 m and barometric pressure 759–760 mmHg). Permission to collect the eggs was granted to DA Crossley by the Minnesota Department of Natural Resources (permit no. 21232). Two eggs from individual clutches were staged to determine age. Incubations lasted no more than 55 days and all eggs were maintained at the sex-determining temperature of 30°C, to ensure all embryos developed as females (Alvine et al., 2013). Eggs were embedded to their midpoint in vermiculite, inside plastic incubators (2.5-l Ziploc© Container, SC Johnson, Racine, WI, United States) that were stored in a walk-in Percival Environmental Control Room (model IR-912L5; Percival Scientific, Perry, IA, United States). The vermiculate was mixed in a 1:1 ratio with water, as previously described (Crossley and Altimiras, 2005).

At approximately 20% development (9–12 days after laying; determined by embryonic staging), eggs were randomly assigned to either normoxic (21% O_2_, partial pressure 152 mmHg) or hypoxic (10% O_2_, partial pressure 72 mmHg) cohorts. The experimental gas conditions were maintained in 76-l Ziplock© bags that were connected to gas supply of either normoxia or 10% oxygen, in the environmental chamber. The normoxic gas was supplied using air pumps (LT 11 Whitewater) passed through a rotameter flow controller. The hypoxic gas was generated using rotameters (Sho Rate Brooks Instruments Division, Hatfield, PA, United States), with compressed N_2_ and air supplied by an air pump (Whisper AP 300 tetra products). Normoxic and hypoxic air was humidified using bubbling chambers and delivered to the bags at a rate of 2–4 L min^–1^. Gas composition was monitored continuously using an oxygen analyzer (S-3AI, Ametek Applied Electrochemistry, Berwyn, PA, United States). Turtle hatch time was between 50 and 52 days, with no difference between experimental groups. Upon hatching, all turtles were housed in common, normoxic conditions (21% O_2_), at 26°C, in a daily 12:12 light–dark cycle, and fed with dry crocodilian food (Mazuri, PMI Nutrition International, Brentwood, MO, United States) two to four times weekly. Mitochondrial experiments were performed when turtles were 8 months old.

### Isolation of Ventricular Mitochondria

Mitochondria were isolated according to previous protocols (Galli et al., 2013, 2016). Turtles were first induced into anesthesia in a sealed plastic box, containing cotton gauze soaked in isoflurane (Isoflo^®^, Abbott Laboratories, North Chicago, IL, United States). Once the turtles were fully anaesthetized, they were euthanized by cranial and spinal pithing. Hearts were removed and weighed, and the ventricle was removed and cleared of connective tissue. The ventricle was then rinsed with ice-cold homogenization buffer (Table 1) and minced into small pieces with a razor blade. The tissue was resuspended in ice-cold homogenization buffer to remove any blood, and the suspension was homogenized in a 12-ml glass mortar using four passes of a loose-fitting Teflon pestle at 120 rpm. The homogenate was centrifuged in polycarbonate tubes at 600 *g* for 10 min at 4°C. The supernatant was filtered through gauze and centrifuged again at 8,500 *g* for 10 min. The pellet was then resuspended in fresh homogenization buffer and centrifuged again at 8,500 *g* for 10 min. Finally, the pellet was resuspended in ∼200 μl of fresh buffer and immediately analyzed for protein content with a Bradford assay, according to the manufacturer’s protocol (Bio−Rad Laboratories, Hercules, CA, United States). The mitochondrial suspension was kept on ice until assayed.

**TABLE 1:** Composition of solutions used for mitochondrial isolation and measuring respiration.

Component	Homogenization buffer	Respiration medium
Sucrose, mmol l^–1^	250	110
HEPES, mmol l^–1^	10	20
EGTA, mmol l^–1^	1	0.5
MgCl_2_, mmol l^–1^		1.4
KH_2_PO_4_, mmol l^–1^		10
K-MES, mmol l^–1^		60
Taurine, mmol l^–1^		20
BSA (fatty-acid-free)	1%	1%
pH	7.4	7.1
Preparation temperature (°C)	4	Room

*All chemicals were purchased from Sigma-Aldrich (St. Louis, MO, United States). pH was adjusted with KOH (5 N).*

### Mitochondrial Respiration and H_2_O_2_ Production

Respiration and H_2_O_2_ production were measured simultaneously with an Oxygraph O_2_-k high-resolution respirometry system (Oroboros Instruments GmbH, Innsbruck, Austria), fitted with an O_2_k-fluorescence LED2-module. Two identical respiration chambers (chamber A and chamber B) were held at the same temperature (25°C) and run in parallel for each experiment. O_2_ electrodes were calibrated every morning with air-saturated respiration medium (Table 1). To measure H_2_O_2_ production, 10 μM Amplex^®^ UltraRed and 1 U ml^–1^ horseradish peroxidase (HRP) were added to each chamber. Amplex^®^ UltraRed oxidizes in the presence of H_2_O_2_ and forms resorufin, using HRP as a catalyst. Amplex^®^ UltraRed was excited at 563 nm and emission was read at 587 nm; 5 U ml^–1^ superoxide dismutase (SOD) was also added to the chambers to convert any extramitochondrial superoxide (O^–^_2_) to H_2_O_2_. At the beginning of each experiment, isolated mitochondria (9.7–29.4 μg protein ml^–1^) were added to each chamber containing 2 ml of respiration medium, and the Amplex UltraRed signals were calibrated with known quantities of exogenously added H_2_O_2_.

### Determining Maximum Respiration Rates and H_2_O_2_ Production Through Individual ETC Complexes

We were interested in measuring three main variables commonly used to assess mitochondrial function. First, Leak respiration rate (Leak) is the amount of O_2_ used to compensate for proton Leak across the mitochondrial inner membrane. Proton Leak can account for up 20–25% of routine metabolic rate in vertebrates, so a reduction in this parameter is a substantial energy-saving mechanism. Second, we measured OXPHOS respiration rate, which is the maximum rate of ADP-stimulated respiration and is an estimate of mitochondrial ATP production. Lastly, we used a protonophore, carbonyl cyanide-*4*-(trifluoromethoxy)phenylhydrazone (FCCP), to uncouple mitochondrial respiration and measure the maximal respiration rate of the electron transport chain, otherwise known as maximum electron-transfer (ET) capacity. We measured these three parameters with different substrate combinations, using a standard substrate inhibitor titration protocol (SUIT protocol), designed according to Pesta and Gnaiger (2012).

**FIGURE 1:**
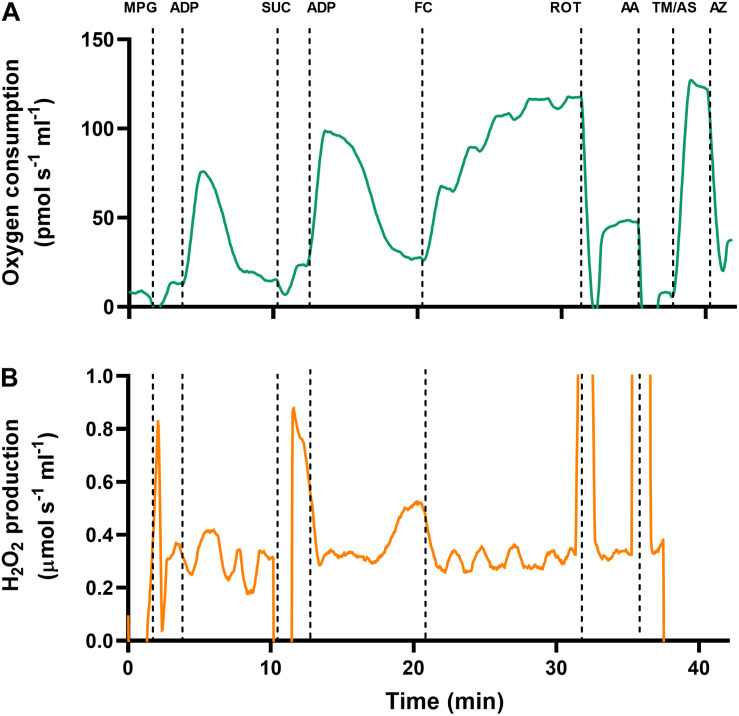
Original traces of simultaneous measurements of **(A)** oxygen consumption and **(B)** H_2_O_2_ production, in turtle ventricular mitochondria. Data are taken from cardiac mitochondria that were isolated from a juvenile turtle previously subjected to developmental hypoxia. Mitochondria were added to the chamber, and a range of substrates and inhibitors were injected to investigate the electron transport chain (see section “Materials and Methods” for full details). MPG, malate, pyruvate and glutamate; ADP, adenosine diphosphate; SUC, succinate; FC, carbonyl cyanide-4-(trifluoromethoxy)phenylhydrazone (FCCP); ROT, rotenone; AA, antimycin-A; TM, N, N,N,N-tetramethyl-p-phenylenediamine (TMPD); AS, ascorbate; AZ, azide.

An original trace of the SUIT protocol is presented in Figure 1. First, pyruvate (5 mM), malate (2 mM), and glutamate (10 mM) were added to achieve Leak respiratory state with complex-I (CI) substrates in the absence of adenylates (Leak_N,CI_). When O_2_ consumption was stable, saturating ADP (5 mM) was injected to activate oxidative phosphorylation with CI substrates (OXPHOS_CI_). Once ADP had been completely phosphorylated to ATP, isolated mitochondria enter the Leak respiratory state in the presence of adenylates with CI substrates (Leak_T,CI_), otherwise known as state-IV respiration. Succinate (10 mM) was then added to assess the additive effects of complex-II (CII) substrates (Leak_T,CI+CII_), and ADP was added again to assess OXPHOS with CI and CII substrates (OXPHOS_CI+CII_). To uncouple mitochondria and assess ET with CI and CII substrates (ET_CI+CII_), FCCP was titrated to a final concentration of 0.1–0.3 μM. Next, the CI inhibitor rotenone (0.5 μM) was added to assess ET_CII_, with CII substrates only. To block the electron transport chain and assess residual non-mitochondrial O_2_ consumption (ROX), the complex-III (CIII) inhibitor antimycin-A (2.5 μM) was added. To assess complex-IV (CIV) activity in isolation, the electron donor N, N,N’,N’-tetramethyl-p-phenylenediamine (TMPD; 0.5 mM) was added in combination with ascorbate (2 mM) to avoid autooxidation of TMPD. Lastly, the CIV inhibitor sodium azide (50 mM) was added to assess background non-mitochondrial O_2_ consumption from the addition of TMPD.

### ROS Production *via* Reverse Electron Transport

Juvenile and adult turtles endure extensive periods of anoxia (up to 4 months) when they overwinter and engage in breath-hold dives (Jackson, 2002). In mammals, one of the main problems leading to anoxic cell death is a burst of mitochondrial ROS from complex I during reoxygenation, driven by the accumulation of succinate (Chouchani et al., 2016). Interestingly, recent work has shown that adult turtles limit oxidative damage at reoxygenation in the heart, by inhibiting the maximal capacity for mitochondrial ROS production *via* reverse electron transport (RET), in addition to limiting the accumulation of succinate (Bundgaard et al., 2019). Therefore, we reasoned that aspects of the RET pathway could be programmed by hypoxia during development, leading to improved cardiac anoxia tolerance in adulthood. To investigate this possibility, we measured H_2_O_2_ production in mitochondria that were incubated with succinate alone. Under these conditions, electrons are transported to the ubiquinone pool *via* CII and subsequently travel in reverse to CI, leading to the production of H_2_O_2_.

### Mitochondrial O_2_ Affinity

Mitochondrial O_2_ affinity was investigated by measuring O_2_ consumption during the transition into anoxia. Briefly, O_2_ concentration in the chambers was reduced to ∼20% of air saturation by blowing nitrogen over the respiration media. The chambers were then closed, the mitochondria were added, and malate, pyruvate, glutamate, and succinate were injected. Saturating levels of ADP were then added and mitochondria subsequently consumed all the remaining O_2_, thus, entering into anoxia, while remaining in the OXPHOS state. To measure mitochondrial O_2_ binding affinity, the partial pressure of oxygen where mitochondrial respiration is half maximal (P_50_) was calculated with Prism software (v9, GraphPad, San Diego, CA, United States), using the equation V_O_2__ = J_Max_⋅[O_2_]/(P_50_+[O_2_]); where V_O_2__ is normalized to O_2_-consumption rate, and J_Max_ is maximal normalized O_2_-consumption rate, and [O_2_] is the concentration of O_2_ in the chamber.

### Data Analysis

Respiration rate and H_2_O_2_ production were normalized to total mitochondrial protein content (Bradford assay, VersaMax spectrophotometer: Molecular Devices, Sunnyvale, CA, United States), and H_2_O_2_ production was also expressed relative to the rate of O_2_ consumption. To estimate mitochondrial efficiency, the respiratory-control ratio (RCR) was calculated as OXPHOS_CI_/Leak_T,CI_, and the OXPHOS coupling-efficiency ratio was calculated as 1-Leak_T,CI_/OXPHOS_CI_. The OXPHOS-control ratio was calculated as OXPHOS_CI+CII_/ET_CI+CII_. We also calculated the ratio of phosphorylated ADP to the atoms of consumed O_2_ (P:O ratio), by making linear extrapolations of O_2_ concentration during OXPHOS (immediately after ADP addition) and Leak_T_ (immediately after ADP depletion), as described by Illingworth; the difference in O_2_ concentration at the intercepts is the total O_2_ uptake. Statistical significances were determined using generalized linear models (GLMs), with sequential Sidak *post hoc* tests, for pairwise comparisons. Developmental O_2_ was the between-group factor, and the pooled mitochondrial samples were the random factors. Data were considered significant when *P* ≤ 0.05.

## Results

### Cardiac Biometry

Exposure to hypoxia during embryonic development decreased body mass in juvenile turtles, while heart mass was unaffected, leading to a larger heart-to-body-mass ratio (Table 2).

**TABLE 2:** Comparison of body and heart masses of juvenile snapping turtles from normoxic (21% O_2_; N21) and hypoxic (10% O_2_; H10) development.

Cohort	Body mass (g)	Heart mass (g)	Heart-to-body-mass ratio (%)
N21	215.1 ± 15.6	0.536 ± 0.042	0.25 ± 0.01
H10	171.3 ± 18.9*	0.529 ± 0.084	0.29 ± 0.02*

Values are means ± standard error of the mean. N-values are 8 each for the N21 and H10 cohorts. *Indicates significant difference between H10 and N21 cohorts, *p* ≤ 0.05.

### Mitochondrial O_2_ Consumption and Basal H_2_O_2_ Production

Mitochondrial preparations were of good quality, as attested by high RCRs (21.5 ± 1.4) and OXPHOS coupling-efficiency ratios (0.95 ± 0.003), with CI substrates (malate, pyruvate, and glutamate). Furthermore, mitochondrial oxygen consumption and H_2_O_2_ production responded to substrates and inhibitors in the expected manner (Pesta and Gnaiger, 2012; Figure 1).

**FIGURE 2:**
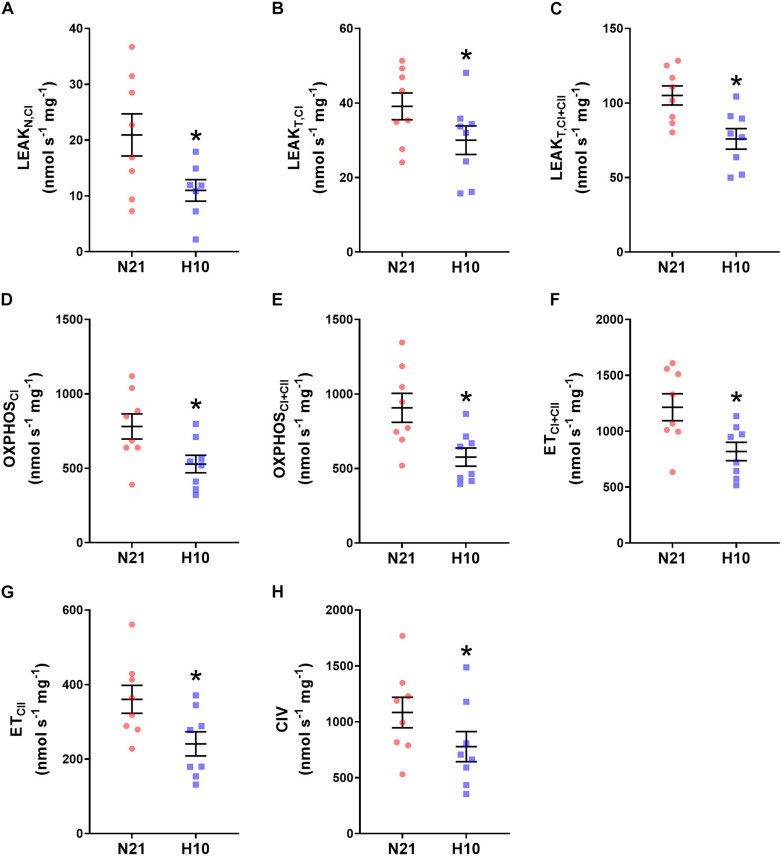
Effect of developmental hypoxia on mitochondrial O_2_ consumption. Mitochondrial O_2_ consumption was measured in juvenile snapping turtles from normoxic (N21, red circles, *n* = 8) and hypoxic (H10, blue squares, *n* = 8) incubations. Each panel represents a respiratory state. **(A)** Leak respiration with substrates for complex I, in the absence of adenylates (Leak_N,CI_). **(B)** Leak respiration with substrates for complex I, in the presence of adenylates (Leak_T,CI_). **(C)** Leak respiration with substrates for complexes I and II, in the presence of adenylates (Leak_T,CI+CII_). **(D)** Oxidative phosphorylation, with substrates for complex I (OXPHOS_CI_). **(E)** Oxidative phosphorylation with substrates for complexes I and II (OXPHOS_CI+CII_). **(F)** Electron-transfer capacity, with substrates for complexes I and II (ET_CI+CII_). **(G)** Electron-transfer capacity, with substrates for complex II (ET_CII_). **(H)** Electron donation to complex IV (CIV). Statistical significance was assessed with individual generalized linear models, followed by Sidak *post hoc* tests, to assess the effect of developmental O_2_ (N21 vs. H10). Values were considered significant when *P* ≤ 0.05, which are denoted by asterisks (*).

**FIGURE 3:**
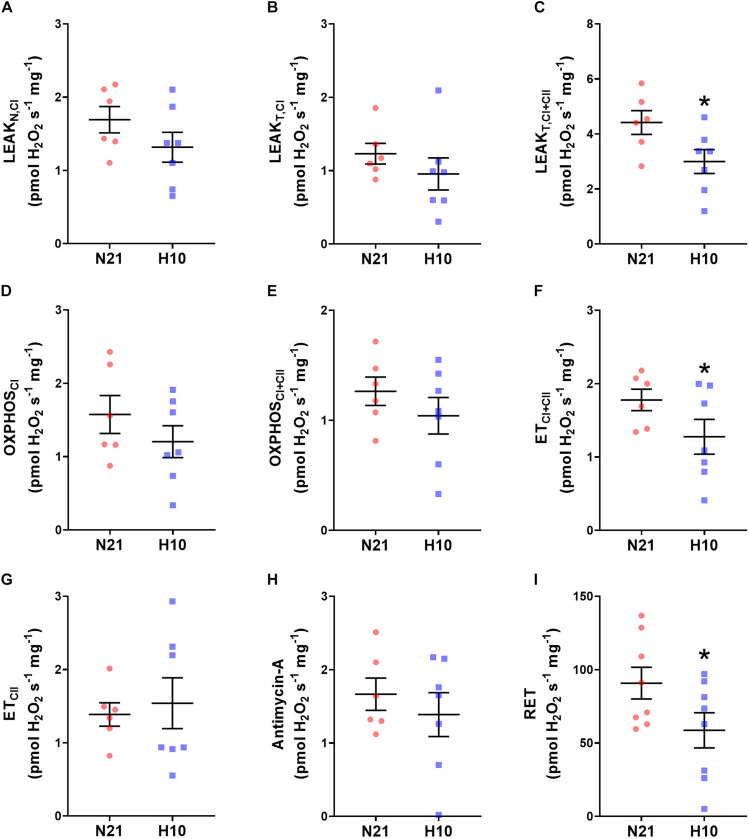
Effect of developmental hypoxia on mitochondrial H_2_O_2_ production. Mitochondrial H_2_O_2_ production was measured in juvenile snapping turtles from normoxic (N21, red circles, *n* = 6) and hypoxic H10 (blue squares, *n* = 7) incubation. Each panel represents a respiratory state. **(A)** Leak respiration with substrates for complex I, in the absence of adenylates (Leak_N,CI_). **(B)** Leak respiration with substrates for complex I, in the presence of adenylates (Leak_T,CI_). **(C)** Leak respiration with substrates for complexes I and II, in the presence of adenylates (Leak_T,CI+CII_). **(D)** Oxidative phosphorylation, with substrates for complex I (OXPHOS_CI_). **(E)** Oxidative phosphorylation, with substrates for complexes I and II (OXPHOS_CI+CII_). **(F)** Electron-transfer capacity, with substrates for complexes I and II (ET_CI+CII_). **(G)** Electron-transfer capacity, with substrates for complex II (ET_CII_). **(H)** Electron-transfer capacity, in the presence of antimycin-A. **(I)** Electron-transfer capacity during reverse electron transport (RET), in the presence of succinate. Statistical significance was assessed with individual generalized linear models, followed by Sidak *post hoc* tests, to assess the effect of developmental O_2_ (N21 vs. H10). Values were considered significant when *P* ≤ 0.05, which are denoted by asterisks (*).

**FIGURE 4:**
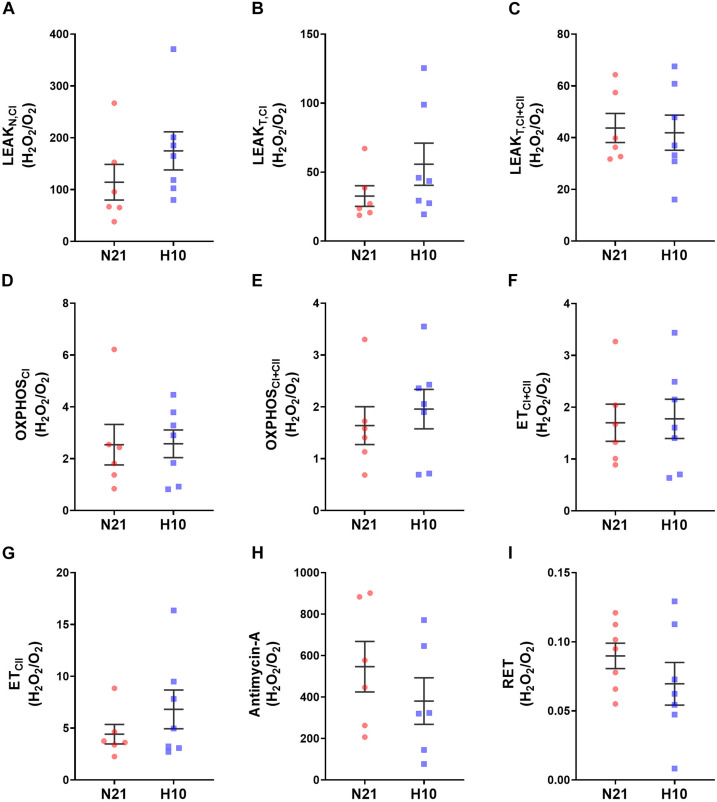
Effect of developmental hypoxia on mitochondrial H_2_O_2_ production, normlalised to respiration rate (H_2_0_2_/O_2_). Mitochondrial H_2_O_2_ production and respiration rate were measured in juvenile snapping turtles from normoxic (N21, red squares, *n* = 6) and hypoxic H10, (blue circles, *n* = 7) incubation. Each panel represents a respiratory state. **(A)** Leak respiration with substrates for complex I, in the absence of adenylates (Leak_N,CI_). **(B)** Leak respiration with substrates for complex I, in the presence of adenylates (Leak_T,CI_). **(C)** Leak respiration with substrates for complexes I and II, in the presence of adenylates (Leak_T,CI+CII_). **(D)** Oxidative phosphorylation, with substrates for complex I (OXPHOS_CI_). **(E)** Oxidative phosphorylation, with substrates for complexes I and II (OXPHOS_CI+CII_). **(F)** Electron-transfer capacity, with substrates for complexes I and II (ET_CI+CII_). **(G)** Electron-transfer capacity, with substrates for complex II (ET_CII_). **(H)** Electron-transfer capacity, in the presence of antimycin-A. **(I)** Electron-transfer capacity during reverse electron transport (RET), in the presence of succinate. Statistical significance was assessed with individual generalized linear models, followed by Sidak *post hoc* tests, to assess the effect of developmental O_2_ (N21 vs. H10). Values were considered significant when *P* ≤ 0.05. There were no statistical differences found between the experimental groups.

Compared to their normoxic counterparts, juvenile snapping turtles previously exposed to developmental hypoxia had significantly lower levels of mitochondrial O_2_ consumption, under all respiratory states and substrate combinations (Figures 2A–H). Basal levels of H_2_O_2_ production were also significantly lower in turtles exposed to developmental hypoxia (Figure 3), but this effect was only statistically significant in two respiratory states: Leak_T_ and ET states, with CI and CII substrates (Figures 3C,F). These latter effects disappeared when H_2_O_2_ production was normalized to O_2_ consumption (Figure 4), suggesting that the lower rates of ROS production in turtles from hypoxic incubations are due to a reduced respiratory rate. We also investigated mitochondrial capacity for H_2_O_2_ production with RET in the presence of succinate alone. Under these conditions, mitochondria from turtles that developed in hypoxia also had significantly lower H_2_O_2_ production (Figure 3I), and this effect disappeared when normalized to O_2_ consumption (Figure 4).

### Mitochondrial Efficiency of ATP Production and O_2_ Affinity

**FIGURE 5:**
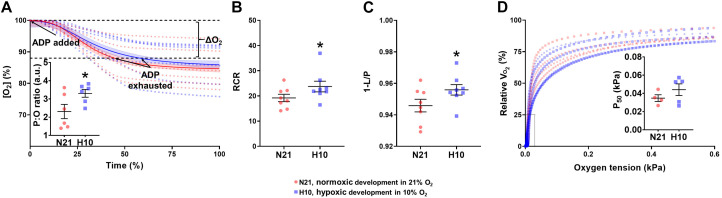
Mitochondrial efficiency of ATP production and O_2_ affinity in juvenile snapping turtles from normoxic (21% O_2_; N21) and hypoxic (10% O_2_; H10) incubations. **(A)** P:O ratio. Oxygen concentration as a function of time is shown for normoxic (*n* = 5) and hypoxic (*n* = 5) developmental groups. Solid lines correspond to the line of best-fit. The inset graph shows the mean P:O values (±SEM). **(B)** The respiratory-control ratio (RCR) and **(C)** OXPHOS coupling efficiency ratio were calculated for normoxic (*n* = 5–8) and hypoxic (*n* = 5–8) developmental groups. Both ratios were measured in the presence of complex-I subtrastes. **(D)** Mitochondrial oxygen affinity. Respiration rate (*V*_*O_2_*_) as a function of oxygen concentration is shown, with individual curves plotted for normoxic (*n* = 5) and hypoxic (*n* = 5) incubation. The inset graph shows the mean P_50_ values ± SEM. Calculations for all variables are given in Section “Materials and Methods.” Statistical significance was assessed with individual generalized linear models, followed by Sidak *post hoc* tests, to assess the effect of developmental O_2_ (N21 vs. H10). Values were considered significant when *P* ≤ 0.05, which are denoted by asterisks (*).

Mitochondria from turtles exposed to developmental hypoxia were significantly more coupled (H10 RCRs were 20% higher than N21) and produced more ATP per molecule of O_2_ (H10 P:O ratios were 30% higher than N21) (Figures 5A,B), indicative of an improved efficiency of ATP production. However, developmental hypoxia had no effect on mitochondrial O_2_ affinity (no change in P_50_) (Figure 5C).

## Discussion

Mitochondria are capable of remarkable phenotypic plasticity in response to hypoxic stress (Galli and Richards, 2014; Murray and Horscroft, 2016; Fuhrmann and Brune, 2017; Sokolova, 2018; Bundgaard et al., 2020). While this kind of remodeling is generally reversible during adulthood, fetal hypoxia can cause permanent mitochondrial abnormalities in mammals, leading to disease susceptibility (Gyllenhammer et al., 2020). Nevertheless, very little is known about the effects of developmental hypoxia on species that routinely experience O_2_ deprivation during embryonic development, such as reptiles. By studying these animals, we can start to identify adaptive vs. pathological responses to developmental hypoxia. In contrast to mammals, here we show that developmental hypoxia can increase the efficiency of ATP production in juvenile snapping turtles, and lower basal ROS production. Interestingly, these responses are very similar to those observed in adult vertebrates that acclimatize to hypoxia (Johnston and Bernard, 1982; Donohoe and Boutilier, 1998; St-Pierre et al., 2000a; Heather et al., 2012; Hickey et al., 2012; Murray and Horscroft, 2016). Therefore, we speculate that developmental hypoxia primes turtle mitochondrial physiology for hypoxic environments in adulthood.

### Developmental Hypoxia Reduces Mitochondrial Aerobic Capacity and ROS Production

Compared to their normoxic counterparts, juvenile turtles that developed under hypoxic conditions had significantly lower rates of mitochondrial respiration. A reduction in aerobic capacity is a common response to chronic hypoxia in a wide range of adult vertebrates (Johnston and Bernard, 1982; Donohoe and Boutilier, 1998; St-Pierre and Boutilier, 2001; Heather et al., 2012; Hickey et al., 2012), including humans acclimatizing to high altitude (Murray and Horscroft, 2016) and animals that are genetically adapted to hypoxia (Ali et al., 2012; Horscroft et al., 2017). While it might seem counterintuitive to reduce aerobic capacity when oxygen is limiting, this strategy is believed to limit ROS production (Murray, 2009; Murray and Horscroft, 2016). ROS production is a major problem in hypoxia, because electrons slip from the chain more often when O_2_ is limited and the chain becomes more reduced (Brand, 2016). Therefore, a lower level of oxidative phosphorylation will reduce the amount of electrons in the electron transport chain and limit the production of superoxide (Murray and Horscroft, 2016). In support of this concept, we also observed reduced basal levels of H_2_O_2_ production in juvenile turtles from hypoxic incubations, and this effect disappeared when H_2_O_2_ was normalized to respiratory rate. Furthermore, developmental hypoxia reduced the rate of turtle H_2_O_2_ production under conditions that promote RET. This finding has ecological significance, because ROS production from RET mainly occurs when tissues are reoxygenated after a period of anoxia or ischemia (Chouchani et al., 2016). Given that turtles regularly engage in long breath-hold dives and overwinter in anoxia for several months, limiting ROS production from RET reduces the likelihood of oxidative stress when turtles resurface. Taken together, our data suggest that developmental hypoxia programs a mitochondrial phenotype in turtles that is better able to cope with oxidative stress.

Although it was not a focus of this study, our experimental design gives some insight into the mechanisms reducing respiratory rate in turtles from hypoxic incubations. Given that the reduction was observed under all respiratory states and substrate combinations, the underlying mechanism is likely to be something fundamental to the entire electron transport chain (Brand and Nicholls, 2011). One possibility is that developmental hypoxia reduced the volume or cristae density of turtle cardiac mitochondria. Indeed, a downregulation of mitochondrial biogenesis is a common response found in adult vertebrates exposed to chronic hypoxia at high altitude (Murray, 2009). Additionally, a widescale reduction in respiration rates can also occur *via* a reduction in the expression or activity of complex IV (cytochrome *c* oxidase), which would limit the respiratory capacity of the entire chain. However, this modification would also increase the reduction state of the electron transport chain, leading to higher rates of ROS production (Dawson et al., 1993). Lastly, several studies have shown that environmental stress during development can epigenetically program mammalian antioxidant-defense systems, which could theoretically alter basal levels of ROS production in adulthood (Strakovsky and Pan, 2012). In this respect, it is interesting to note that hypoxia tolerance is closely related to antioxidant capacity in ectothermic vertebrates (Bickler and Buck, 2007), including turtles (Storey, 1996). Clearly, further experiments are necessary to confirm the mechanism that program respiratory rate and ROS production in turtles from hypoxic incubations. Future research should be directed toward investigating the effects of developmental hypoxia on turtle mitochondrial morphology with electron microscopy, as well as the expression or activity of electron-transport-chain complexes and antioxidants. Furthermore, it would be interesting to measure the activity of anaerobic enzymes, such as lactate dehydrogenase, which are known to be altered by developmental hypoxia in some ectotherms (Matschak et al., 1998; Ton et al., 2003; Anderson and Podrabsky, 2014).

### Developmental Hypoxia Increases the Efficiency of Mitochondrial ATP Production

In order to offset the reduced aerobic capacity that accompanies chronic hypoxia, many adult animals increase the efficiency of mitochondrial ATP production by reducing proton leak and increasing the P:O ratio (Gnaiger et al., 2000; Semenza, 2007; Zhang et al., 2013; Murray and Horscroft, 2016). Interestingly, we found that developmental hypoxia programmed these same attributes in turtle ventricular mitochondria. The P:O ratio is a measure of the number of ATP molecules synthesized by oxidative phosphorylation for each oxygen atom reduced. Although oxygen consumption is routinely used as a proxy for ATP production, the amount of ATP generated per unit of oxygen consumed can vary significantly between individuals, and it is well established that environmental stress can alter this parameter (Salin et al., 2015). Improvements in the P:O ratio can have major implications for animal life-history traits, including growth, reproduction, and lifespan (Barros et al., 2004; Stier et al., 2014; Salin et al., 2015). While several factors can influence the P:O ratio, the most common mechanism that accounts for individual variation is proton leak. This phenomenon refers to the process whereby protons leak across the mitochondrial membrane without the generation of ATP (Divakaruni and Brand, 2011). Importantly, proton leak can account for up to 25% of an animal’s metabolic rate, so a reduction in this parameter can greatly increase the efficiency of ATP production. Therefore, the elevation in the P:O ratio we observed in turtles from hypoxic incubations could be driven by a decrease in proton leak. In support of this contention, we found that developmental hypoxia significantly lowered levels of leak respiration, leading to an increase in mitochondrial coupling (higher RCR values). Interestingly, we found the same result in juvenile alligators exposed to developmental hypoxia (Galli et al., 2016), suggesting that proton-leak pathways are a common target for developmental programming. Future research should, therefore, determine the mechanism driving the reduction in proton leak, which could include changes to the inner mitochondrial membrane, like the composition of the lipid bilayer, expression of the adenine nucleotide translocase, and/or suppression of uncoupling proteins (Zhao et al., 2019). Nevertheless, it should be noted that a decrease in proton leak in the absence of other changes will lead to an increase in ROS (Brand, 2000).

### Developmental Hypoxia Has No Effect on Mitochondrial Oxygen Affinity

Several studies have shown that mitochondrial P_50_ is lower in hypoxia-tolerant vs. hypoxia-sensitive species (Scott et al., 2011; Zhang et al., 2013; Lau et al., 2017), and it can be decreased in some adult vertebrates after acute and chronic episodes of hypoxia (Gnaiger et al., 1998b; St-Pierre et al., 2000b). A low P_50_ allows the mitochondria to extract oxygen from the cytosol more effectively, which is particularly important during periods of hypoxia, when the concentration gradient for O_2_ has been reduced (Gnaiger, 2001). Several mechanisms can account for these differences, including changes in the abundance and activity of complex IV (the site of oxygen reduction), altered flux through the electron transport chain relative to complex-IV capacity, and differences in the mitochondrial membrane potential (Bienfait et al., 1975; Verkhovsky et al., 1996; Gnaiger et al., 1998a). Nevertheless, in the present study, we did not find any differences in mitochondrial O_2_-binding affinity between the developmental cohorts.

### Physiological Significance and Perspectives

Adult vertebrates acclimatizing to hypoxic environments undergo mitochondrial remodeling to enhance oxygen delivery, maintain ATP balance, and limit oxidative stress. The mechanisms underlying this remodeling include a reduction in aerobic capacity and proton leak, a reduced basal ROS production, and an increase in the P:O ratio. For the first time, we show that these same attributes can be permanently programmed in turtles by exposure to hypoxia during embryonic development. The molecular mechanisms underlying this response remain to be investigated, but they could include hypoxia-inducible factor (HIF) signaling. Indeed, HIF-1 activation is known to coordinate mitochondrial remodeling in adult vertebrates exposed to hypoxia (Murray and Horscroft, 2016; Thomas and Ashcroft, 2019), and this pathway is activated in hypoxic zebrafish embryos, leading to enhanced hypoxia tolerance in adulthood (Robertson et al., 2014). In addition, mammalian mitochondria can be programmed during development *via* epigenetic alterations to the nuclear and mitochondrial genomes, changes to the mitochondrial–telomere axis, and the accumulation of mitochondrial DNA defects (Gyllenhammer et al., 2020). Whatever the mechanism, we suspect that mitochondrial programming in turtle embryos will improve hypoxia tolerance in adulthood, which would be beneficial for breath-hold diving and overwintering in anoxic environments (Jackson, 2000). Indeed, our recent study suggests turtles from hypoxic incubations have improved cardiac anoxia tolerance (Ruhr et al., 2019). Therefore, developmental hypoxia could represent an important environmental cue for turtles that primes their physiology for a future in low-oxygen environments.

## Data Availability Statement

The raw data supporting the conclusions of this article will be made available by the authors, without undue reservation.

## Ethics Statement

The animal studies were carried out according to the approved animal care protocol of the University of North Texas Institutional Animal Care and Use Committee No. 1403-04.

## Author Contributions

IR and GG: conceptualization, methodology, formal analysis, and writing—original draft. IR, GG, JC, and DC: investigation. GG and DC: resources, supervision, and project administration. IR, GG, and DC: writing—review and editing and funding acquisition. IR: visualization. All authors contributed to the article and approved the submitted version.

## Conflict of Interest

The authors declare that the research was conducted in the absence of any commercial or financial relationships that could be construed as a potential conflict of interest.
